# Supplementary material for: Are carbon emissions trading and green financial instruments synergistic? -Comprehensive quantitative research based on content analysis
Source: PLoS One. 2024 Mar 7;19(3):e0298601. doi: 10.1371/journal.pone.0298601 (PMC10919601; doi:10.1371/journal.pone.0298601)
Supplement: S1 Data — (ZIP) [file pone.0298601.s001.zip › Data/地级市Z_F工作报告.zip!/║■─╧/│╗╓▌╩╨/└┤╫╘╣1⁄2╓┌║┼í╛┬φ┐╦╩2╛▌═°í┐_2013.docx]

各位代表：

现在，我代表市人民政府向大会报告工作，请予审议，并请市政协各位委员及其他列席人员提出意见。

一、2012年政府工作回顾

2012年是非同寻常、富有成效的一年。面对世界经济复苏明显放缓和国内经济下行压力加大的严峻形势，在市委的坚强领导下，全市上下按照“大干新三年，再创新辉煌”的总体要求和“一化两城三创四大”的总体思路，科学应对、攻坚克难，较好地完成了市四届人大一次会议确定的年度目标任务。预计（下同），地区生产总值1530亿元，同比增长12%以上；财政总收入182亿元，一般预算收入120亿元，分别增长20.8%和26%；城镇居民人均可支配收入20300元，农民人均纯收入7350元，分别增长15.5%和18%；万元GDP能耗下降4%，化学需氧量、二氧化硫、氮氧化物、氨氮排放量分别下降2%、1.6%、2%和2.2%。桂阳县、永兴县进入全省县域经济十强县，嘉禾县迈入全省县域经济发展先进县。

——项目建设在破解瓶颈中取得新成效。围绕实施“十二五”投资和项目建设“836”计划，融资240亿元，增长11.1%；报批项目用地近3万亩，增长1.7倍，较好破解了资金、土地等项目建设瓶颈制约。固定资产投资1095亿元，增长35%。交通大建设、产业大转型、城市大提质强力推进，实施投资5000万元以上主要建设项目890个，完成投资620亿元，纳入省项目建设“三个一”行动计划的51个项目投资271.8亿元。交通大建设投资123.6亿元，厦蓉、衡武2条高速公路通车，高速公路通车里程由150.6公里增加到476.7公里；衡茶吉铁路安仁段基本完工；5条154公里干线公路基本通车，381公里干线公路开工建设，郴永大道春节前可基本拉通路基。金旺仙溪总厂、富士产业园一期、奥美森工业园、中化弘源氟化工、宏润茶油加工及基地建设、风力发电等392个产业项目，郴州国际会展中心、郴江路延伸段、郴北和桂东220千伏输变电站等243个城市基础设施项目，市体育中心体育馆、市第一人民医院门诊综合楼、市中医院搬迁主体工程、市人防疏散指挥基地、中央下放煤矿棚户区改造、东江水库移民避险搬迁安置等95个民生和社会事业项目建成或完成年度投资计划。储备重大前期项目203个，计划总投资2811亿元；桂阳桃源水库完成项目前期工作并通过国家烟草局援建资金审查，宜章莽山水库项目获国家发改委批复立项，桂宁、临连高速和西气东输郴州段等重大项目前期工作取得突破性进展。

——产业素质在转型升级中实现新突破。工业经济质量提高。规模工业增加值增长15.5%，其中高加工度工业、装备制造业、电子信息产业增加值分别增长22%、35%和32%。新增年产值过10亿元的企业5家。新认定高新技术企业11家，高新技术产业增加值增长30%。园区规模工业增加值增长24%，占全市规模工业的比重提高4个百分点。郴州有色产业园升级为省级高新区，申报国家级高新区工作进展顺利；省级安仁工业集中区获批。新增金旺、湘江、宇腾、金贵、济草堂、永兴冰糖橙6件中国驰名商标，品牌战略实现历史性突破。煤矿企业加快兼并重组，组建煤业集团10个。永兴县获批国家“城市矿产”示范基地。现代服务业加快发展。社会消费品零售总额555亿元，增长15%。步步高、美美世界、苏仙福地和友阿国际广场等城市综合体开工建设，建成标准化农家店770家。金融机构各项存款余额突破1300亿元，各项贷款余额570亿元，分别增长16.7%和23.4%；新增贷款105亿元。保险业保费收入25.2亿元。郴州农村商业银行加快组建，新增村镇银行3家、融资性担保公司7家。市城投二期债券成功发行，三湘菌业股权挂牌交易，金贵银业上市成功过会，新增5家重点上市后备企业；湖南有色金属（郴州）价格指数发布，南方稀贵金属交易所在全国发布现货交易价格。房地产开发投资110亿元，增长18%。“旅游项目建设攻坚年”活动任务全面完成，成功举办第二届国际休闲旅游文化节，接待游客2348.7万人次，旅游总收入154.5亿元，分别增长15.3%和19.5%。公路水运客货运周转量、邮电业务量分别增长28.9%和14.4%。现代农业稳步发展。粮食生产实现8连增，总产186万吨。改造中低产田10.3万亩，新建高产稳产粮田4万亩、专业菜地1万余亩，实施农业标准化生产面积220万亩，创建国家级畜禽标准化示范场3个。汝城县成为全国农业综合开发高标准农田建设示范县，安仁县成为全国油茶产业发展示范县。收购烟叶104.9万担。农产品加工业产值增长24%，农产品加工产值与农业产值比为1.2:1。新增农民专业合作社465家，增长56.5%，带动农户26.6万户。培育五星级农庄15家。推广新型农机具6.1万台套。

——开放承接在抢抓机遇中迈出新步伐。承接产业转移“四千工程”积极推进，湘南承接产业转移示范区建设首批21个重大项目进展顺利。建成标准厂房360万平方米，出租率95%，承接产业转移项目370多个。郴州国际会展中心被省政府明确为湘南唯一的承接产业转移会展中心。铁路口岸即将开放，国际快件业务恢复，铁路物流中心一期投入使用，开放平台不断完善。招商引资成效明显，与央企对接合作不断深化。实际利用外资7.5亿美元，到位内资270亿元，分别增长19%和19.5%；外贸进出口总额25亿美元，加工贸易进出口总额16亿美元，分别增长60%和2.5倍。重点领域和关键环节改革全面推进。国企改革扫尾工作步伐加快，国有资产监管实现全覆盖。公务卡制度改革强力推行。资源税改革试点进展顺利。郴州经开区与北湖区实现“区区合一”。乡镇区划调整和机构改革全面完成，农、林、水体制改革深入推进。医药卫生、文化、科技体制和价格改革取得新成效，事业单位分类改革有序推进。宜章县列入国家资源可持续发展改革县，嘉禾县龙潭镇、宜章县白石渡镇列入全国第三批改革发展试点镇。

——城乡面貌在统筹发展中发生新变化。郴资桂-郴永宜“大十字”城镇群区域规划和郴资桂两型社会示范带总体规划纲要、综合配套改革方案、新农村建设布局规划等加快编制，罗霄山片区四县区域发展与扶贫攻坚规划取得重大突破。中心城区以“三创”为抓手，推进“十大提质”和“五化工程”，实施基础设施项目155个，完成投资103亿元。改造背街小巷18条，完成“城中村”改造项目13个，拆除违法建筑25.3万平方米、违规户外广告和门店招牌6万平方米。新建改造公厕54座、垃圾站15座。“351111”工程进展顺利。数字城管正式运行。新增新能源环保型公交车140台。规划审批投放停车位5.29万个。郴资桂两型社会示范带建设第一阶段任务全面完成，实施重大建设项目455个，完成投资450亿元，郴州大道两旁建筑完成“平改坡”，郴州到资兴城际公交开通。县城、中心镇加快扩容提质，汝城县热水镇、三江口镇和苏仙区望仙镇、资兴市黄草镇、桂东县清泉镇等特色小城镇建设全面推进。创建市级新农村建设示范村19个、省级2个。“点亮郴州”行动积极推进，“十城万盏”半导体照明工程全面实施，推广半导体照明路灯1.75万盏，农村安装路灯1.7万盏；完成村庄整治208个，其中39个农村环境连片整治示范村通过省验收。农村公路建设完成路面511公里。农网改造升级工程涉及359个行政村，受益农户9.53万户。新一代天气雷达投入试运行。水利建设投资31.6亿元，江源水库大坝竣工，除险加固病险水库178座，治理中小河流74.8公里，新增灌溉面积26.7万亩，解决了28万农村人口饮水不安全问题。东江湖列入国家“一湖一策”试点工程。苏仙区、宜章县、临武县列入全国小型农田水利重点县，桂阳县、桂东县列入国家河塘整治试点项目县。生态建设强力推进，全市森林覆盖率提高到64.75%。“三年城乡绿化攻坚”开局良好，创建国家森林城市正式启动，造林绿化投资53亿元，荒山荒地造林绿化70.4万亩，公路绿化4072.5公里，河流水渠绿化537.4公里，水库四周绿化1.4万亩，创建省级生态乡镇9个、生态村33个。中心城区“绿城攻坚”二期任务超额完成，新建改造公（游）园15个，建成区绿化覆盖率、绿地率、人均公园绿地面积分别达到38.31%、36.21%和9.53平方米，申报国家园林城市通过省初审。“十大环保工程”全面启动，淘汰落后产能125万吨，39个环境污染综合整治项目进展顺利，25个重金属污染治理项目中13个主体工程完工、其余进展顺利，苏仙区矿山地质环境治理项目成为国家示范工程。

——社会事业在协调推进中有了新进步。“民生100工程”99项目标任务全面完成，财政直接用于民生的支出增长14.6%。新增城镇就业6.3万人、农村劳动力转移就业5.28万人，城镇登记失业率3.96%，荣获首批“全国创业先进城市”称号。城镇职工医疗保险、城乡居民医疗保险、生育保险实现市辖区市级统筹，农村五保户和孕产妇在县乡医疗机构住院、分娩基本费用全免。11个县市区全面启动新农保和城居养老保险试点，基本实现养老制度全覆盖。新增居家养老试点社区24个，改扩建乡镇敬老院29所。城乡低保对象月人均补助分别达到229元和100元。新农合参合率保持100%，住院费用平均报销比例76.3%。新开工保障性安居工程住房7.01万套（户）。6万贫困人口实现脱贫。教育强市建设持续推进，创建义务教育合格学校107所，完成中小学校舍安全工程53个，改造农村义务教育薄弱学校130所，新增义务教育学位中心城区近万个、县城2万个；利用闲置校舍改建公办幼儿园79所，农村中小学、教学点增设幼儿班489个；郴州师范学校升格创建工作有序推进，郴州职业技术学院创建省级示范性高职院校通过立项，郴州技师学院获评全国高技能人才培训基地，湘南学院稳步发展。科技创新能力增强，有4个项目列入国家级科技计划、2个高新技术产品列入国家重点新产品计划；获专利授权677项，实施专利项目693项；永兴县跻身国家知识产权强县。基本公共卫生服务均等化步伐加快，11类41项公共卫生服务全面落实，国家基本药物制度延伸到村卫生室、覆盖率97.4%，区域卫生信息列入国家试点城市，食品药品城乡一体化监管体系基本建成。成功举办“相约郴州”-海峡两岸昆曲交流展演活动和第六届郴州市艺术节，新建农家书屋776个，提质改造和免费开放乡镇综合文化站250个，农村放映免费电影3.5万场。文明创建活动深入开展。人口计生工作“基层基础建设年”活动扎实有效，生育关怀行动惠及贫困计生家庭10万人次，人口自然增长率6.57‰。创建体育之城有序推进，郴州籍运动员易思玲伦敦奥运会勇夺首金。物价总体平稳，新建平价商店39家。广播影视、新闻出版、民族宗教、外事侨务、法律援助、统计、贸促、海关、检验检疫、档案、地震、水文、科普、地方志、爱卫、慈善和工会、妇女、儿童、老龄人、残疾人等事业取得新进步。驻郴部队和国防后备力量为地方发展作出新贡献。公路站点超限超载率降至0.09%以下。“限摩规电”持续推进，客运管理服务明显改善。安全生产形势总体稳定，没有发生较大以上事故。推进社会管理创新，加强应急管理和社区建设，完善村（居）民自治，在全国首设县级司法局长任“首席人民调解员”，有效化解了矛盾纠纷，解决了群众合理诉求。深化“打黑除恶”等专项行动，社会大局和谐稳定。公众安全感及干部队伍建设群众满意度居全省第二。

——政府公信力在强化执行中得到新提升。围绕提升政府公信力，全面清理行政审批项目，行政许可事项精减19%；巩固“两集中、两到位”改革成果，进驻市政务中心的行政审批、政务服务事项423项，并全部授权到位，窗口按时办结率99.9%，审批时限提速50%以上；县市区全面完成“两集中、两到位”改革并建立政务中心，199个乡镇（街道）建成便民服务中心。市级网上处罚和电子监察系统应用进一步规范；县市区全部建成网上审批和电子监察系统，并与市级互联互通。政府采购、工程建设招投标、国土资源交易项目纳入市公共资源交易中心，交易额50亿元；所有乡镇建立农村公共资产资源交易中心。健全市政府领导接听“市长公开电话”、接待信访群众并督办落实等工作机制，市长公开电话群众来电办结率、群众满意率分别为96.67%和92.8%。加强审计监督、财政投资评审和预算绩效管理，审计核减投资4亿多元，市政府部门单位向市人大常委会报告审计问题整改情况逐步常态化。大力加强公务员队伍建设，扎实开展作风大整顿，作风建设得到中央领导和社会各界的关注与肯定，干部队伍干事创业、建功立业激情迸发。全面推进政府法律顾问制度。认真执行市人大及其常委会决议决定，坚持重大事项向人大报告、向政协通报，自觉接受人大和政协监督，注重发挥各民主党派、工商联、无党派人士和人民团体参政议政作用。办理市人大代表建议275件、市政协提案288件，办复率100%。

各位代表！即将过去的2012年，我们在复杂严峻的经济形势中实现了良好开局，在矛盾交织的转型发展中迈出了坚实步伐，在聚精会神的真抓实干中赢得了广泛支持！这些成绩的取得，是省委、省政府和市委正确领导的结果，是各级人大、政协监督支持的结果，更凝聚着全市人民的智慧和汗水。在此，我代表市人民政府向全市各族人民，向人大代表、政协委员，向各民主党派、工商联、无党派人士和人民团体，向驻郴解放军、武警和消防官兵，向中省驻郴单位，向离退休老同志，向所有关心支持郴州发展的各界朋友，表示衷心的感谢并致以崇高的敬意！

回顾一年来的工作，我们也清醒地看到发展中存在的困难和问题：产业结构还不合理，大企业大项目不多，自主创新能力较弱，部分企业生产经营困难，转型发展任务繁重，经济增长下行压力较大；财政减收增支因素增多，城乡区域统筹发展、居民持续增收困难较大，民生保障还处于较低水平；城市建设和管理还存在薄弱环节；一些领域和地方安全生产、环境保护、社会治安形势依然严峻；行政效能和干部作风建设还有待进一步加强，等等。对此，我们将采取有效措施，切实加以解决。

二、2013年主要工作任务

2013年是全面贯彻落实党的十八大精神的开局之年，是为全面建成小康社会奠定坚实基础的重要之年，也是我市“大干新三年，再创新辉煌”的关键之年。一方面，要看到宏观经济形势的复杂性和不确定性，我市转型发展的艰巨性和紧迫性，及早谋划、提前部署，把困难估计更足一点、把工作部署更细一点、把措施制定更实一点；另一方面，更要看到宏观经济形势总体趋好，我市发展格局出现重大变化，发展机遇前所未有，发展态势保持强劲。只要我们坚定信心、抢抓机遇、科学应对，就一定能够乘势而上、掌握主动、大有作为。政府工作的总体要求是：深入学习和全面贯彻落实党的十八大精神，按照“大干新三年，再创新辉煌”的总体要求，以“一化两城三创四大”为总揽，以提高经济增长质量和效益为中心，以改革创新为动力，深入推进“四化两型”，扩内需、调结构、提质量，重生态、惠民生、促和谐，努力实现经济稳中求进、又好又快发展和社会和谐稳定。主要预期目标是：地区生产总值增长12%以上；财政总收入和一般预算收入均增长15%；固定资产投资增长28%；社会消费品零售总额增长15%；城镇居民人均可支配收入、农民人均纯收入分别增长15%和17%；城镇登记失业率控制在4.5%以内；居民消费价格涨幅控制在4%左右；人口自然增长率控制在7.6‰以内。重点抓好七个方面的工作：

（一）着力扩大国内需求，努力实现经济持续健康发展。继续发挥投资对经济增长的关键作用，更加注重消费拉动作用。促进投资稳定增长和结构优化。深入实施“十二五”投资和项目建设“836计划”，持续推进交通大建设、产业大转型、城市大提质，力争固定资产投资1400亿元以上。安排总投资1亿元以上重大建设项目764个，年度计划投资1021亿元，其中基础设施项目195个，年度计划投资264亿元，力争炎汝高速基本建成，23个408公里干线公路项目全部完成路基工程并建成通车100公里，桂宁、临连高速公路开工建设，郴州国际会展中心、郴江河综合治理、郴州城际客运站、桂东沤菜水库等项目竣工，南岭大道南段、西气东输郴州段、城市管道燃气建设和供水设施提质改造等项目加快推进。产业建设项目283个，年度计划投资398亿元，力争台达产业园二期、格兰博智能机器人、丰越多金属综合回收、桂阳富氧炼铅等项目建成投产，富士产业园、奥美森工业园、资五产业园、三一产业园、宜章氟化学工业园和“5080”煤化工、汝城中湘钨业、桂阳工业园有色冶炼加工项目区等项目加快推进。节能环保项目56个，年度计划投资80亿元，力争华润脱硝改造工程一期、雷坪矿区重金属污染治理等项目竣工，东江湖生态环境保护试点工程、苏仙区金属矿区矿山地质环境治理工程、永鑫资源综合利用等项目加快推进。民生和社会发展项目230个，年度计划投资279亿元，力争城东新区居民安置房建设、临武文化体育广电中心等项目竣工，市城投大厦、城镇生活垃圾处理设施、国有工矿棚户区改造等项目加快推进。强力推进项目建设。加强项目储备、包装和推介，加大争资立项力度，做好郴州机场、桂永郴赣铁路等重大项目前期工作。积极争取信贷支持，努力扩大民间投资，加快发展私募基金，开展民间资本登记金融服务中心试点，创新融资工具和模式，力争全市融资200亿元、市城投三期债券和汝城小水电债券发行。抓好土地征储、供应工作，优先保障重点产业、民生工程和重大基础设施项目用地需求。完善重大项目联动协调、督查督办和考评问责机制，加强项目全程质量、安全和工程监理，优化项目建设环境，确保工程进度和质量。强化政府投资项目全程监管和跟踪审计。加快培育消费新增长点。进一步提高居民消费能力，积极实施居民收入倍增计划，完善收入分配制度，加大对改善民生、扩大消费的财政性支出，增加对城乡低收入群体补贴，全面提高城乡居民特别是低收入群众收入。进一步拓宽社会消费领域，积极开拓农村消费市场，着力培育住房、汽车、旅游等消费热点，提升家政物业、医疗保健、文化娱乐等服务消费，发展网络购物、信贷消费、租赁消费等新兴消费业态，有效引导“产地消费”。进一步改善城乡消费环境，开展美食节、汽车展、商品展、房交会等主题促销活动，加强市场监管和消费维权，保持市场价格总水平基本稳定，坚决打击商业欺诈、制假售假等行为，让消费者安全、放心消费；大力实施“市场建设三年行动计划”，继续推进“万村千乡市场工程”和农贸市场改造提质，加快步步高、美美世界、苏仙福地和友阿国际广场等城市综合体和专项市场、便民超市建设；积极推进国家再生资源回收体系综合试点城市和农产品现代流通综合试点城市建设。

（二）着力推进转型升级，加快构建现代产业体系。深入实施产业转型发展“三年行动计划”，力争工业技改投资增长26%，新增规模工业企业40家，培育产值过30亿元的企业5家、过50亿元的2家，规模工业增加值增长15.5%。加快传统产业转型升级。开展“改造提升传统产业年”活动，推进冶炼行业兼并重组，突出发展有色金属精深加工，积极发展矿业总部经济，力争国家有色贵重金属质检中心在郴建设，重点支持宇腾有色、金贵银业、华信有色、金旺铋业和中国五矿、云南锡业在郴企业等优势企业发展。加快发展石墨精深加工，重点支持南方石墨等优势企业发展和北湖隐晶质石墨深加工等项目建设，加快建设全国最大的微晶石墨制品生产基地。大力发展化工新材料、盐（氟、硅）化工、精细化工，加快氟化工产业园建设，重点支持郴州氟化学、中化弘源、中盐华湘、郴化集团等优势企业发展。巩固发展火电、小水电产业。深入推进煤矿企业兼并重组，组建30个年产30万吨以上煤业集团。积极开发特种水泥新品种，重点支持南方水泥、华新水泥、祥丰建材等优势企业发展。加快烟叶生产及深加工基地建设，重点支持天泰复烤搬迁改造、郴州卷烟厂异地技改工程建设。力促新兴产业突破发展。加快湖南数字视讯产业（郴州）基地建设，重点支持高斯贝尔、台达电子、晶讯电子、炬神电子等优势企业发展，延伸产业链，形成电子信息优势企业集群。以有色金属、萤石和石墨资源为依托，重点开发先进储能材料、先进复合材料、高性能金属结构材料、基础材料，大力发展新材料产业。重点支持奥美森、富士电梯、春意空调、郴州粮机等优势企业发展，扶持嘉禾江南铸都建设，加快发展先进装备制造业。重点推进华磊光电、格瑞普、天塘山风电场等项目建设，积极开发利用新能源。着力发展“三废”治理产业和环保产业，把郴州高新区、永兴循环经济示范园打造成环保产业基地。加大重点领域工业软件研发和应用力度，扶持发展软件和信息服务新型业态。力争战略性新兴产业增加值增长20%，高新技术产业增加值增长25%以上。促进现代农业快速发展。加强耕地保护，推进高标准农田建设和中低产田改造，建设高产稳产粮田2.7万亩，力争粮食总产达到190万吨。加强“菜篮子”基地建设，新增专业菜地1万亩。实施农业标准化生产面积240万亩，新建生猪标准化规模养殖场50个，力争创建省级以上畜禽标准化示范场8个。突出抓好以郴桂嘉为重点的现代烟草农业示范区、郴资桂-郴永宜现代农业示范带建设，新建现代农业示范园区11个，收购烟叶110万担以上。大力实施农业产业化“12345”工程，重点支持舜华鸭业、湘粮裕湘、宏润油业、邦尔泰油脂、东江湖渔业、玲珑王茶叶、生平米业等龙头企业发展，加快建设资兴罗围、桂阳芙蓉、临武城东等农产品加工园区。扶持联户经营、专业大户和家庭农场，大力发展农民专业合作社联合社，创新农业社会化服务机制。新增农民专业合作社500家，入社农户达到18%以上。积极发展休闲农业，培育一批国家级星级农庄、全国休闲农业示范项目和全国魅力乡村。开展无公害农产品、绿色食品、有机食品和地理标志产品认证。农村土地流转500万亩以上。推广新型农机具5.6万台套。做好防汛抗旱、动植物防疫等工作。加速园区经济规模扩张。充分发挥园区在项目集中、企业集聚、产业集群、发展集约中的示范引领作用，使各类园区成为推动经济发展的主力军，力争园区规模工业增加值增长23%。实施“产城融合”战略，努力形成“以产兴城、以城促产、产城一体”的园区发展新路子。加速园区提档升级，支持苏仙、桂东工业园区升级为省级园区，支持符合条件的省级园区申报国家新型工业化示范基地，力争郴州高新区升格为国家级高新区、资兴经开区申报为省级高新区、郴州经开区2-3年内创建为国家级园区。引导园区产业、企业加强协作配套，增强产业配套能力，推动产业集群发展。完善园区仓储、物流、商务服务及生活配套功能。推动服务业提质增量。突出发展生产性服务业，力争生产性服务业增加值增长13%以上。促进金融与产业深度融合，组建郴州农村商业银行，推进县域农信社改革，优化金融生态环境，力争新增贷款100亿元以上；大力推进企业上市，力争新增上市公司2家；规范发展非银行金融机构，帮助解决中小企业融资难题。加快发展现代物流业，积极发展电子物流、冷链物流、供应链物流，重点扶持惠尔物流中心、桂阳商贸物流中心、资兴鸿通仓储等项目和湘南国际物流园、临武凌峰物流园等园区建设，着力引进1-2家大型专业物流龙头企业。积极发展研发设计、咨询评估、商务会展、文化创意等专业服务业，加快国家高技术服务产业基地建设。加大资源整合力度，推进“数字郴州·智慧城市”建设，提高全社会信息化水平。大力发展消费性服务业。继续推进旅游“五个一”工程和旅游精品项目建设，完善功能配套，强化整体营销，办好第三届国际休闲旅游文化节，游客人数、旅游总收入分别增长16%和20%以上，力争东江湖成功创建国家5A级旅游景区。合理调控房地产供应总量和结构，加大普通商品房供应，注重提高住房品质，促进房地产市场长期平稳健康发展。支持发展养老、健康、家政、物业等民生服务业。强化科技创新驱动发展。大力推进国家知识产权示范培育城市创建，加大高端人才引进培养使用力度，完善政府主导、企业主体、产学研结合的科技创新体系，加快实施一批重大科技专项，推动科技深度融入经济社会发展主战场。引导创新要素向企业集聚，鼓励企业增加研发投入，重点培育5-10家科技创新型骨干企业。推动科技和金融深度融合，完善政府科技投入机制，引导社会资金投向高新技术企业和创新项目。重点抓好郴州中南大学技术转移中心、亚欧水资源研究和利用中心郴州分中心、郴州国家生产力示范中心、郴州战略性新兴产业孵化基地等创新平台建设，积极筹建郴州应用技术研究院，力争组建4-6家市级技术研发中心、创建1家省级科技创新平台，早日实现郴州国家级研发中心“零”的突破。出台《关于加快推进创新型郴州建设的意见》，支持县市区实施科技服务与创新能力提升“三年行动计划”。加强知识产权保护，力争新增中国驰名商标4件以上。

（三）着力深化改革开放，增强经济发展内生活力和动力。围绕推进湘南承接产业转移示范区建设，更加注重体制机制创新，更加注重先行先试，把全社会的智慧和力量凝聚到创新驱动发展上来。深入推进重点领域改革。持续开展“推进改革年”活动，力争在8大领域40多项改革上取得新突破。完成事业单位分类改革，推进机关后勤服务社会化改革。深化城管体制改革，推进城管重心下移和管护作业市场化。加快户籍制度改革，有序推进农业转移人口市民化。深化市城区财政体制、部门预算、非税收入管理和公务卡制度改革。稳妥推进资本市场、资源性产品价格等要素市场改革。完善国有资产监管机制。深化医药卫生体制改革，重点推进县级公立医院、医疗保险付费方式等改革。深入推进农村土地、林业、水利等综合改革，创新农业生产经营体制机制。加快承接产业转移。加大政策争取和落实力度，拓展放大政策创新联动效应，引领全省新一轮改革创新，争创全国承接产业转移示范市，力争郴州出口加工区升级为综合保税区。继续推进标准厂房建设，新建标准厂房350万平方米。加快铁海联运二期建设和铁路口岸开放，开通“五定班列”；做大国际快件业务总量，发展快件物流；启动郴州口岸国际物流中心和电子口岸建设，加快构建口岸大通关体系。创新招商引资方式，突出产业招商、要素招商和以商引商，加强对国内重点企业、重点地区的定向招商，提高招商活动和招商队伍绩效。力争实际利用外资8.6亿美元、到位内资320亿元，分别增长15%和18.5%，新引进一批投资过10亿元的重大项目。努力扩大对外贸易。支持宇腾有色、金贵银业、金旺铋业、台达电子、高斯贝尔等进出口优势企业扩大进出口总量、优化进出口结构，加快培育重点出口企业集群。加强特色出口基地建设，扩大自主研发、自主品牌、自主营销产品出口，保持传统市场份额，深度开发新兴市场。加快发展服务贸易和服务外包，积极创建承接国际服务外包基地城市。力争外贸进出口总额增长20%，其中加工贸易增长50%。大力支持非公经济发展。鼓励、支持和引导非公经济加快发展，落实“非禁即入”投资原则，拓宽民间投资领域，激发各类市场主体发展活力，力争非公经济增加值增长13.5%。充分发挥民营企业生力军作用，出台扶持政策，加强服务平台建设，引导民营企业加快制度、科技和管理创新，提高企业综合竞争力。全面落实扶持中小微企业政策措施，帮助企业减轻负担；建立小微企业创业基地，加快郴州高新区大学生创新创业园建设，推进小微企业转型升级，力争三年内培育100户专精特新小微型标杆企业、20个地方特色产品成为国家和省名牌产品、10户具有发展潜力的“小巨人”计划企业成为大型企业。

（四）着力推进新型城镇化，促进城乡发展一体化。以城乡规划一体化为引领，坚持速度与质量并重，完善中心城区功能，加快城镇群培育、新农村建设和贫困地区发展，统筹城乡区域协调发展。增强提升中心城区综合承载力。按照建设国际化现代化开放城市的要求，进一步优化城市设计，增强规划的前瞻性和约束性。加快城市综合交通、电力、燃气、医疗卫生、体育设施等专项规划编制，做好下湄桥西片区控规修编、东河组团北片区等片区控规编制和重大项目布局规划，力争控规覆盖率95%。安排城市基础设施项目150个，年度计划投资180亿元。围绕“三创”，继续推进城市建设管理“十大提质”，重点抓好54条道路及配套设施建设，强力推进城中村、棚户区、市场和街景改造，加快地下管网、污水处理和环卫、燃气、石油、电力设施建设，城区污水处理率80%以上。启动东江引水二期工程。加快推进“351111”工程，打造十大精品楼盘、十大精品公共建筑和十大精品市政工程，提升城市品位。整合提高数字城管功能，大力推进精细化管理。围绕创建国家交通管理模范城市，优先发展公共交通，加快城区智能交管服务系统建设；规划审批停车位3.37万个并逐步建成投放，着力缓解出行难、停车难和交通拥堵问题。实施生态水环境建设，倾力打造山水名城。推进“绿城攻坚”三期，实施“12234”园林精品工程，推进城区园林化、城郊森林化、道路林荫化、庭院花园化，力争建成区绿化覆盖率44%以上、绿地率38%以上、人均公园绿地面积10.8平方米以上，成功创建国家园林城市。强力推进“大十字”城镇群和郴资桂两型社会示范带建设。完成《郴州“大十字”城镇群区域规划（2012-2030）》编制，完善郴资桂两型社会示范带区域各专业专项规划，做好郴州大道沿线乡镇和村庄规划编制工作，北湖、苏仙两区在全市率先完成乡镇总体规划编制。确保郴永大道主体工程完工，启动京珠高速耒宜段提质改造工程；力争X090线主体工程完工，加快X091线提质改造，构筑郴资桂第二通道；加快苏仙区望仙镇、桥口镇、良田镇和北湖区石盖塘镇、华塘镇及桂阳县正和镇等卫星镇建设，促进资兴、桂阳、永兴、宜章融城发展，培育特色鲜明的郴资桂-郴永宜“大十字”城镇群。郴资桂两型社会示范带继续推进“十大工程”建设，建立健全示范带建设联席会议制度和考核评价机制，力争在发展两型产业、统筹两型城乡、共建两型生态、营造两型氛围、探索两型体制机制等五大领域取得新突破；实施总投资1亿元以上重大项目400个，年度计划投资550亿元；力争东江湖保护项目纳入省两型社会示范建设重点。统筹推进城乡区域发展。出台加快推进城乡发展一体化的意见，建立健全统筹城乡发展的财政、土地、投融资、社会管理等体制机制，实施发展特色县域经济强县工程，加快形成以工促农、以城带乡、工农互惠、城乡一体的新型工农、城乡关系。坚持规划先行、突出特色、产业优先、注重生态、多方投入，大力推进小城镇建设。突出县城和中心镇扩容提质，全面完成县城总体规划修编，加快建设水、电、路、讯及污水处理等城镇基础设施。支持嘉禾省级城乡一体化示范县建设。大力推进特色小城镇建设，重点加快乡镇“八个一”建设，抓好30个重点示范镇建设。全面推进新农村建设，实施“十城百镇千村”新农村建设工程，市级重点抓好郴州大道新农村示范带建设，每个县市区重点抓好1个试点镇连片带动5个以上自然村的新农村建设工作。创建省级新农村建设示范村3个以上，新增市级新农村建设示范村10个以上。全面推进“点亮郴州”行动和“十城万盏”半导体照明工程，深化农村环境综合整治，重点整治200个主要交通干线沿线村庄。开展“水利攻坚年”活动，力争投资40亿元，重点抓好桂阳桃源、安仁茶安、宜章莽山等水库建设和180座病险水库除险加固、5条33公里中小河流治理、77处33万人农村饮水安全工程等水利基础设施建设，争创“国家水生态文明建设试点市”。积极实施农业综合开发、烟水配套工程、土地综合治理等项目。完成农村公路路面建设300公里。加大农网改造力度。进一步细化并加快实施罗霄山郴州片区区域发展与扶贫攻坚规划，创新扶贫机制，大力争资立项，推进罗霄山片区106国道示范带建设，促进贫困地区跨越发展。全面推进大中型水库移民避险解困工作，启动移民新型农村社区建设试点，扶持移民地区加快发展。

（五）着力加强生态文明建设，努力建设美丽郴州。树立尊重自然、顺应自然、保护自然的生态文明理念，把生态文明建设融入经济建设、政治建设、文化建设、社会建设各方面和全过程，提升郴州永续发展能力。积极创建国家森林城市。贯彻《绿色湖南建设纲要》，实施《郴州市国家森林城市建设总体规划》，加快“大十字”城镇群绿化、环东江湖生态保护和治理、生态脆弱区提质改造等七大工程建设。继续推进“三年城乡绿化攻坚”，荒山荒地造林绿化41.5万亩，封山育林40万亩，低产林改造30万亩；干线公路绿化和绿化提质改造1038公里，农村公路绿化1430公里；河流水渠绿化593公里；53个示范乡镇和200个示范村完成村镇绿化。强力推进封山育林，严格实行“五禁”，保护森林资源、古树名木和野生动物。鼓励发展苗圃生产，苗木自给率和乡土树种使用率80%以上。大力开展义务植树和绿色单位创建活动，力争全民义务植树尽责率80%以上、群众对“创森”知晓率和支持率90%以上。全面推进十大环保工程。严格奖惩机制，确保完成十大环保工程年度目标任务。突出抓好重金属污染治理，实施东河、西河和武水河等小流域重金属治理整体推进示范工程，依法关闭、淘汰和改造升级一批涉重金属企业，加强矿山地质环境恢复治理，争创一批省级以上生态县、生态乡镇和生态村。实施主要水系出境断面水质目标管理，推进国家重点生态功能区建设，保护好东江湖水环境。大力推进节能减排。坚持调结构、控增量、减存量，大力发展循环经济，加快电机节能、余热回收节能技改、垃圾发电等节能工程和火电、水泥行业低氮燃烧及烟气脱硝工程建设，全面推进工业企业排污权交易，严格控制新建“两高一资”项目，加快淘汰落后产能。加大清洁能源推广力度，中心城区取缔燃煤锅炉60余台、新增新能源环保型公交车100台，争创省级低碳交通示范城市；农村建设户用沼气池6500口，推广太阳能热水器2500台、省柴节煤灶1500台。全面完成省下达的节能减排任务。

（六）着力保障改善民生，维护社会和谐稳定。完善保障和改善民生的长效机制，促进基本公共服务均等化，不断提高全市人民的幸福指数。继续推进“民生100工程”。在更宽领域用更高标准推进“民生100工程”，促进城乡居民生产生活条件持续改善。重点在劳动就业、社会保障、学前教育、医疗卫生、科技文化、食品药品安全、扶贫移民开发、农村基础设施、“三农”保险等方面实施民生项目，让发展成果更多更公平地惠及全市人民。力争民生工程投资100亿元以上，财政直接用于民生的支出增长18%。稳定扩大就业规模。巩固全国创业先进城市创建成果，大力推进创业带动就业三年行动计划，健全创业带动就业“五大体系”，构建和谐劳动关系，以全民创业实现更高质量的就业。统筹做好高校毕业生、农村转移劳动力、就业困难人员和退役军人就业。加强公共就业服务体系建设，强化就业和职业技能培训，重点保障工业园区企业用工需求，职业技能培训10万人次。新增城镇就业5.2万人、农村劳动力转移就业4.5万人，城镇“零就业”家庭实现动态清零。健全完善社会保障体系。稳步推进新农保工作，积极开展城乡居民大病保险试点，适时启动建筑领域工伤保险，继续开展农房统保。做好社会保障卡发放工作，全面推进城镇居民医保、新农合、城乡医疗救助体系建设，城镇职工、居民医保参保率巩固在95%以上，新农合参合率巩固在98%以上。进一步提高农村五保户分散供养标准和城乡低保水平。实施“空巢老人”关爱行动，完善高龄补贴制度，推进居家养老服务，完善信息化养老服务平台；加快苏仙区老年福利中心建设，力争实现县县有一所福利院，新增日间照料中心床位和机构养老床位3000张；加强乡镇敬老院建设管理。完善保障性住房建设、分配、管理和退出机制，稳妥推进公租房建设，新建（筹建）保障性安居工程住房4.6万套（户）。统筹发展社会事业。坚持教育优先发展，强化义务教育均衡发展，继续推进中心城区和县城义务教育扩容，建设义务教育合格学校38所，实施中小学校舍安全改造项目50个；加快发展学前教育和特殊教育，规范和扶持发展民办教育，大力发展现代职业教育，积极发展继续教育和终身教育；支持郴州师范升格创建、郴州电大创建郴州开放大学、郴州职业技术学院建设省示范性高职院校、郴州技师学院和湘南学院加快发展，打造人才培养高地；着力构建覆盖学前教育到高等教育的家庭经济困难学生资助体系；加强教师队伍建设，提高教育教学质量。强化基本公共卫生服务，健全农村三级医疗卫生服务网络和城镇社区卫生服务体系，巩固完善基本药物制度；完善重大疾病防控机制，加快区域卫生信息化建设，做好重大疾病防控工作；鼓励发展中药材产业，扶持发展中医药事业。完善食品药品城乡一体化监管体系，切实保障食品药品安全。深入开展健康教育活动。完善公共文化服务体系，加快实施文化项目建设“双十工程”，大力发展新闻出版、广播影视、文学艺术事业，扎实推进文化强市建设。深入推进文明创建活动。广泛开展全民健身运动，推进体育之城创建。坚持计划生育基本国策，强化人口计生基层基础工作，加大计生奖扶和综合治理力度，完善独生子女伤残死亡家庭扶助机制，促进人口计生工作上水平。认真组织第三次全国经济普查。积极支持工会工作。大力发展妇女儿童事业。加强青年工作和未成年人保护。加快残疾人无障碍设施和康复中心建设。加强防灾减灾体系建设，提高气象、地质、地震灾害防御能力。切实做好民族宗教、外事侨务、统计、贸促、海关、检验检疫、档案、科普、水文、地方志、爱卫、慈善和老龄人等工作。全力维护社会稳定。完善突发公共事件应急管理机制，加强基层社会管理和服务体系建设，强化企事业单位、人民团体在社会管理和服务中的职责，加快形成源头治理、动态管理、应急处置相结合的社会管理机制。健全基层民主自治，加快城乡社区建设，夯实社会管理基础。推行网格化服务管理模式，突出抓好流动人口、特殊人群、非公经济组织的服务管理。畅通信访渠道，推进领导“大接访”、信访“积案化解”工作常态化，发挥行政复议和仲裁作用，健全以人民调解为主的矛盾纠纷调处机制，及时化解信访积案、解决群众合理诉求。完善网上舆论引导机制，及时回应网民关切。深化“平安郴州”建设，健全打防控管一体化的群防群治网络，强化治安重点地区排查整治，深入开展“打黑除恶”等专项行动，着力解决群众关心的治安热点、难点问题，进一步提升群众安全感和满意度。深入推进公路治超和客运管理服务大提质。突出抓好企业主体责任和政府监管责任落实，推进企业安全标准化建设，持续开展“打非治违”等专项行动，加强校园周边环境整治和校车安全管理，坚决杜绝重特大事故发生。加强国防建设和国防教育，支持驻郴部队、国防后备力量和人民防空建设。

（七）着力加强政府自身建设，不断提升政府公信力。面对科学发展的艰巨任务，政府工作必须更有作为；面对人民群众的殷切期盼，政府工作人员必须付出更大努力。不断提升科学发展能力。大力简政放权，推动政府职能向创造良好发展环境、提供优质公共服务、维护社会公平正义转变，为市场主体提供良好发展环境。更加注重加强社会管理和公共服务，支持基层创造性开展工作，增强发展的协调性和惠民的公平性。完善重大决策听证、风险评估和责任追究机制，加强宏观经济形势研判，推进政府决策科学化、民主化和制度化。不断提升依法行政能力。认真执行市人大及其常委会决议决定，健全社会主义协商民主制度，自觉接受人大和政协监督，优质高效办理建议提案。认真听取和采纳各民主党派、工商联、人民团体和社会各界意见，主动接受社会公众和新闻舆论监督。健全政务公开机制，推进行政权力和公共服务网上公开运行。健全政府常务会议学法制度，加强政府法律顾问工作。规范行政处罚自由裁量权，促进公正文明执法。全体政府工作人员必须切实增强学法尊法守法用法意识，绝不允许以言代法、以权压法、徇私枉法。不断提升执行落实能力。巩固深化行政审批制度改革，完善网上政务服务、电子监察系统和市、县、乡、村四级政务服务体系，将行政审批、行政处罚、公共资源交易等公共服务和公共管理事项纳入网上政务服务系统办理。全面推行便民服务免费代办制度，年内所有村（社区）建立便民服务代办点。整合市县两级社会求助服务平台，提高市长公开电话办结率。完善综合绩效管理和评价机制，精简会议、文件、接待，把更多精力放在化解矛盾、推进工作上，把有限资源和财力放在发展经济、改善民生上。政府及其工作人员必须坚持实干富民、实干兴业、实干兴邦，敢于实践、敢于创新、敢于担当；继续开展作风大整顿，落实中央“八项规定”和省委“九条规定”，坚决克服形式主义和官僚主义。不断提升廉洁从政能力。全面加强清廉政府建设，严格执行廉洁从政规定，坚持用制度管权管事管人。推进节约型机关建设，严格控制机构编制，降低行政成本。强化对重要领域、重点岗位的行政监察和审计监督，坚决查处违法违纪案件，始终保持惩治腐败的高压态势，以良好的政府形象赢得人民群众的信任和拥护。

各位代表！新的一年，推进科学发展的任务更加繁重，造福郴州人民的使命更加光荣。让我们高举中国特色社会主义伟大旗帜，在中共郴州市委的正确领导下，团结和依靠全市人民，求真务实，开拓创新，为加快建设国际化现代化开放城市和全面建成小康社会而努力奋斗！
